# Evaluation of a Pilot: Inspection Facilitation and Collaboration Using a Mixed Reality Device

**DOI:** 10.1007/s43441-023-00594-2

**Published:** 2023-11-22

**Authors:** Peter Baker, Tamika Cathey, Jared R. Auclair

**Affiliations:** 1 Live Oak Quality Assurance LLC, Austin, TX USA; 2 TDC & Associates Consulting, Montgomery, AL USA; 3 Northeastern University, 360 Huntington Ave, Boston, MA 02115 USA

**Keywords:** GMP, Inspection, Virtual, Regulatory

## Abstract

The COVID-19 pandemic forced industry and national regulatory authorities (NRAs) to think about innovative ways to ensure business continuity, including Good Manufacturing Practices (GMP) inspections. Even prior to COVID-19, it was understood that GMP site inspections, especially redundant inspections, are a time and resource-intensive process for both industry and regulators in high-income countries and often prohibitive to resource-challenged countries. Thus, we investigated the use of a mixed reality device and Microsoft (MS) Teams as a platform for mixed reality (hybrid) remote inspection. This pilot involved a mock GMP inspection of a drug manufacturing facility in the United States. The mock inspection was conducted by two former USFDA (US Food and Drug Administration) investigators, facilitated by representatives from Northeastern University along with The Bill and Melinda Gates Foundation. Also participating in the inspection were inspectors from national regulatory agencies (NRAs) from the African continent, including Nigeria, South Africa, Uganda, and Zimbabwe, and representatives from the Pre-Qualification Inspection Unit at the World Health Organization (WHO). Harmonized inspectional guidance from PIC/s (GMP Guide) and WHO (TRS 823) were used as the standards for conducting the mock inspection. We found that mixed reality, used in conjunction with a collaborative text messaging system, is a viable tool to facilitate remote inspections and allows inspectors participating remotely to write their own independent inspection reports.

**Supplementary Information:**

The online version contains supplementary material available at 10.1007/s43441-023-00594-2.

## Introduction

Currently, legislation in most jurisdictions requires GMP inspections for regulated medical products. For example, in the United States, 21 CFR Parts 210 and 211, cGMP (current Good Manufacturing Practice) in Manufacturing, Processing, Packaging, or Holding of Drugs and Finished Pharmaceuticals, is the code of regulations [1]. To ensure product quality, GMP inspections are usually conducted by GMP inspectors from each country in which a product manufacturer applies for authorization for the sale of their drug product [2]. Often inspectors from the NRA (national regulatory agency) in the country of manufacturing origin for the product will also participate. For example, if a product is produced in India and the manufacturer applies for authorization for sale in the United States, US FDA (United States Food and Drug Administration) inspectors inspect that plant. If that same product also applies for authorization for sale in Europe, Australia, and Japan, each of those jurisdictions generally sends inspectors to the manufacturing site to perform independent GMP inspections unless there is a Mutual Recognition Agreement (MRA) in place with a country that has already inspected the relevant manufacturing site for the relevant product [3]. A global pandemic straining the supply chain and travel restrictions due to fluctuating COVID-19 guidelines increased the risk of non-quality assured products being shipped and sold throughout the world, which was further exacerbated by a lack of MRAs or other facilitating agreements between resource-challenged, lower-income countries and better-resourced nations, [3, 4]. Because there are restrictions on sharing commercial, confidential, and trade secret information, it is often difficult for regulatory agencies to share unredacted inspection reports with other inspectorates, causing redundancy in inspectional activities [5].

Thus, accelerated by the COVID-19 pandemic, regulatory authorities around the world began to seriously consider the advantages of remote audits or inspections. In fact, the European Medicines Agency (EMA) released a document, *Guidance on remote GCP inspections during the COVID-19 pandemic*, in May of 2020 and the US FDA released a draft document, *Conducting Remote Regulatory Assessments Questions and Answers Draft Guidance for Industry*, in July of 2022 [6, 7]. In addition, the European Directorate for the Quality of Medicines & HealthCare (EDQM) will recommend the use of Real-Time Remote Inspections (RTEMIS) as an option in the future [8].

However, because of the accelerated nature in which remote inspections were implemented very few pilots have been conducted to highlight both the strengths and weaknesses of the technology and approach. Thus, a pilot study was conducted with the goal of identifying the benefits and challenges of remote inspections using a mixed reality device. Mixed reality, different from augmented or virtual reality, is the combination of the physical and digital world [9]. In theory, mixed reality devices along with meeting platforms, such as Microsoft Teams, should allow for real-time, onsite GMP inspection of a facility to be conducted simultaneously with virtual interactions and assessments being performed by participating global inspectorates/inspectors. Overall, this pilot program identified areas in which such an approach worked, and areas of challenge related to virtual real-time GMP inspections.

## Materials and Methods

The mock inspection was performed at Hikma Pharmaceuticals USA Inc in Columbus, Ohio on March 16th, 2022, to identify challenges and future opportunities for the use of mixed reality to help perform virtual GMP inspections. The mixed reality headset used for this study was the Microsoft Hololens2, with the following features and specifications: 4-GB LPDDR4x system DRAM, holographic density > 2.5 K radiants, 802.11ac (2 × 2) wireless, Bluetooth 5.0, 2k 3:2 light engines resolution, 52° diagonal/43° horizontal/29° vertical field of vision, 566 g, eye tracking, head tracking, hand tracking, Windows Hello Authentication, and 6 degrees-of-freedom (6DoF) tracking (Microsoft Corporation).

The mock inspection was conducted by two former US FDA Investigators (co-authors, Cathey and Baker) and facilitated by representatives from Northeastern University (principal investigator) and The Bill and Melinda Gates Foundation. Harmonized inspectional guidance from PIC/s and WHO (TR 823) were used as the standards for conducting the mock inspection [10, 11]. The mock audit was observed by multiple inspectors from NRAs from the African continent (referred to as NRA observers): (in no particular order) the South African Health Product Regulatory Authority (SAHPRA), Nigeria’s National Agency for Food & Drug Administration & Control (NAFDAC), the Medicines Control Authority of Zimbabwe, the National Drug Authority of Uganda, and representative(s) from the World Health Organization’s Pre-qualification Team Inspectorate. The observers were able to view the headset user’s field of vision and provide verbal directions highlighting key areas of focus, ask inspectional questions, make verbal requests to the on-the-ground host inspectional team, and provide written feedback via MS Teams which could be seen virtually by the headset wearer. At the end of the mock audit, NRA observers took notes as they would if they were performing the audit in person, documenting their experiences as feedback to support the pilot study conclusion outcomes.

### Pre-pilot Inspection

Representatives from Northeastern University, The Bill and Melinda Gates Foundation, and the two US FDA alumni inspectors met virtually with Hikma Pharmaceuticals representatives’ several weeks before the initiation of the mock GMP inspection to identify site resources. These identified site resources included Subject Matter Experts (SMEs) and technical meeting platforms that would be used during the audit. The manufacturing facility had hosted a previous virtual audit and had tested its internet connectivity in various GMP areas throughout the facility. In these initial conversations it was determined we would focus our mock inspection on the quality laboratory, GMP manufacturing suites, and the shipping/receiving area, which included stability chambers.

The day prior to the mock inspection, Northeastern representatives and the two US FDA alumni inspectors met, set-up the HoloLens2, performed practice runs, and adjusted the auditing plan accordingly. Briefly, we set-up the HoloLens2 to bypass the retina scan, which allowed any user to unlock the system. Each inspector spent an hour using the HoloLens2 to adjust to the spatial resolution, hand tracking and general operation of the headset, in particular how to use the Remote Assist (Microsoft Corporation) program. During this practice run, the individuals not wearing the mixed reality headset gave directions to the HoloLens2 operator who was then able to perform the task.

### Pilot Inspection Day

On the day of the inspection, the two US FDA alumni inspectors performing the inspection, Northeastern and Hikma representatives were on-site in a conference room to observe via Microsoft Teams and representatives from the national regulatory authorities mentioned above and The Bill and Melinda Gates Foundation were observing from remote locations around the world using Microsoft Teams. Hikma’s Lead Quality team hosted the facility tour and department area subject matter experts were interviewed by the inspectors as they would have been during a traditional audit. During the mock inspection, both of the US FDA alumni inspectors wore the HoloLens2 to provide two different user perspectives. In addition to the two US FDA alumni inspectors, the Northeastern Principal Investigator also wore the HoloLens2 to facilitate the mock inspection as he had the most experience with the headset. The inspector wearing the HoloLens2 was considered the “lead” or primary inspector. The speakers of the HoloLens2 were placed on mute and the microphone was enabled for the best audio reception.

The inspector not wearing the headset was responsible for monitoring the connection and quality of projection of the Inspection through the personal connected device (e.g., smartphone or tablet) and facilitated the participation of those participating via MS Teams. Questions were provided to the on-site inspectors in real-time via the MS Teams chat function through the mobile device. At pre-defined intervals, audio conversations with all participating inspectors were held (enabling microphones/speakers as appropriate). At the conclusion of the mock inspection, Hikma’s on-site audit participants were interviewed for feedback regarding the successes, challenges, and potential next steps for how the pilot program could be used from a regulated facility perspective.

### Post-pilot Inspection

The two US FDA alumni inspectors and Northeastern University Principal Investigator interviewed all NRA and WHO observers via one-on-one interviews and a survey. Participants were asked to provide feedback on the feasibility of the technology as a tool giving the observing inspectors the ability to write their own, independent, GMP inspection reports. In addition to this individual feedback, participants were asked to fill out an online survey (Supplemental Figure S1). Participants were given 7 days to respond to the survey and were reminded twice via email to do so.

## Results

A one-day mock inspection of a small molecule generics manufacturing site was performed by two US FDA alumni inspectors and a Northeastern University faculty. The HoloLens2, when connected to a strong Wi-Fi connection, provided a clear-high resolution view of the on-site GMP areas, even in distances that were not in direct line of site. Areas captured by the device could be pinned and held for future evaluation during the inspection. Inspectors could also circle and highlight areas of interest in which virtual observers/NRAs could annotate any potential non-conformance and/or observation. Accessing the internet through a wireless hotspot was unsatisfactory, providing intermittent reception and images of poor quality. Inspectors were able to develop and maintain inspection reports based on internal procedures with real-time visual assessments. Thus, not relying on final reports from other NRAs that may not meet internal/national standards or fit into existing procedures or that have redactions, which makes the documents less helpful.

## Discussion

### FDA Alumni Inspectorate Perspective

This pilot demonstrated the feasibility of conducting remote collaborative and interactive GMP audits using a mixed reality device in conjunction with interactive text messaging. During the mock inspection, observers from multiple NRAs could interact directly with the on-site inspectors, asking specific inspectional questions, evaluating the facility, and reviewing requested documents, which allowed a remote regulatory assessment (RRA) to be conducted.

The US FDA has shown increased interest in remote assessments to help achieve oversight of regulated industries. This interest is demonstrated by the US FDA *Draft Guidance for Industry: Conducting Remote Regulatory Assessments* released in July 2022*.* The draft guidance includes new language highlighting “remote interactive evaluations” even when oversight activities are conducted by parties other than US FDA (e.g., state and foreign regulatory partners). The guidance also places emphasis that US FDA may conduct RRAs (e.g., live streaming) during these oversight activities*.* One additional benefit noted during the pilot program was the ability to use mixed reality to train new inspectors rapidly and effectively through real-time observation of the inspection process.

Also, it was noted by the FDA alumni inspectors that additional training on the mixed reality headset was necessary for them to be able to perform a remote audit efficiently. Also, several technological challenges were identified (e.g. wi-fi connectivity, headset battery life, etc.). That said, the technology can be used to hedge interruptions caused by a global pandemic, natural disaster, and other unstable situations allowing for inspectional continuity and consolidation of limited resources.

### NRA Perspective

A survey was used to gather information from the NRAs who participated in the mock inspection. We had an 83% response rate to the survey (5 out of 6 participants). The majority of the NRA participants that responded (3 of the 5) said that the use of mixed reality was a useful tool for inspections (supplemental information). In general, the respondents appreciated the ability to collaborate with other inspectors and stated that the technology had the potential to reduce the regulatory burden of NRAs conducting individual GMP inspections. When asked about dislikes related to the mixed reality tool participants mentioned challenges with wi-fi connectivity, the need for robust training to use the headset, and the lack of battery life. Lastly, participants said they could use the information gathered from the remote inspection to write their own inspection report or partially write their own inspection report. The inspectors mentioned a pre-onsite inspection review of documents/reports (e.g., one or two weeks prior to the onsite audit) would have facilitated the writing of their own GMP reports along with the virtual inspection; in this study we did not include the pre-review document inspection. Alternatively, the onsite inspectors could review documents during the inspection and provide feedback to inspectors observing remotely.

In one-on-one interviews with the participants, these same themes were observed. Briefly, the participants highlighted the value of technology to reduce regulatory burden, improve GMP inspection harmonization, and provide an opportunity for resource-limited countries to perform their own GMP audit. Participants mentioned the utility of the technology as a training tool for new inspectors, mentioning that the technology was not only appropriate for GMP inspections but also inspection of QC (quality control) labs. In addition, it was recommended that a pre-audit readiness check be performed several weeks prior to the onsite inspection. Finally, several of the NRAs had participated in virtual audits where a company representative leads the audit using remote technologies (e.g., cell phone camera), but the NRAs preferred our approach in which a regulatory inspector (or two) would be on site to facilitate the inspection and not the company itself.

### Industrial Partner Perspective

At the conclusion of the mock inspection, the company (sponsor) participants were interviewed for feedback regarding the pilot study. The primary feedback was the use of mixed reality technology could be greatly beneficial to the firm’s ability to host future remote audits or assessments with other health authorities. Also, the technology could be used to support foreign supplier qualifications/verifications where logistical challenges have arisen in the past to ensure better compliance oversight. The firm was interested in the possibility of leveraging the technology to facilitate remote training courses and/or support FDA’s remote regulatory assessments.

The mixed reality technology allows for better-resourced countries to participate in an increased number of GMP inspections, allowing for better utilization of human and financial resources, decreased redundancy of inspections, and further alignment between countries on approaches to and processes for inspections. The mixed reality technology allows for less well-resourced countries to participate in GMP inspections where they might not otherwise be able to do so. It removes the need for travel to manufacturing sites to perform an audit, thus eliminating travel costs and time spent traveling and on-site. It provides an opportunity for regulatory resource sharing and collaboration in the inspection process while still allowing each country to write their own GMP reports based on their own observations and their own national regulations and make their own regulatory decision regarding the findings.

Thus, there is a significant opportunity for the use of mixed reality devices in remote inspections. However, in-depth training is needed to ensure the headset wearer is able to use the headset efficiently. In addition, Wi-Fi connectivity is not consistent throughout most manufacturing facilities, which must be considered. Thus, participating inspectors (including those joining remotely) must discuss the procedures to follow if a loss of Wi-Fi signal occurs during the inspection. When connection is restored, it is recommended that a concise summary of events that occurred during the loss of connection be provided by the inspection lead. The lead inspector will not know when the connection is lost; this function is performed by the inspector with the mobile device.

In addition, historically, remote inspections are scheduled and have not been used for unannounced inspections. In many of these instances the remote aspect of the inspection is facilitated by an employee of the company manufacturing the drug, thus they need to be scheduled in advance to facilitate this interaction. However, in the case where the inspector performing the remote audit is from a national regulatory authority, such as in this pilot, it is possible that unscheduled remote inspections are possible.

Review of printed or electronic documentation was also a challenge using the mixed reality headset, but it is a crucial part of the audit as it can identify data integrity issues, for example [12]. We recommend requesting and reviewing copies of documentation and audit trail reports for instrumentation before the inspection.

This can be done by requesting copies of documentation and audit trail reports for instrumentation prior to the inspection. In addition, documentation can be reviewed by the onsite auditor wearing the headset in real time or after the onsite inspection. Inspectors can share their findings using the principles of regulatory reliance [13], eliminating the need to set up multiple mutual recognition agreements (MRAs) between different NRAs.

## Conclusion

A mixed reality device, used in conjunction with a collaborative text messaging system, allowed multiple NRAs to perform a regulatory inspection of a GMP facility simultaneously, while still following their respective internal procedures. Remote inspections will reduce the number of onsite inspections any company may have to host, by effectively allowing multiple regulatory agencies to inspect simultaneously. Thus, the use of a mixed reality device and MS Teams platform to perform a GMP inspection including multiple NRAs is feasible. Further testing of improved versions of the technology will provide a more seamless experience for inspectors on-site and participating virtually.

### Supplementary Information

Below is the link to the electronic supplementary material.

Supplemental Figure 1: Regulators Questionnaire (PDF 275 kb)
