# Supplementary material for: Evaluation of a Pilot: Inspection Facilitation and Collaboration Using a Mixed Reality Device
Source: Ther Innov Regul Sci. 2023 Nov 22;58(1):11–5. doi: 10.1007/s43441-023-00594-2 (PMC10764403; doi:10.1007/s43441-023-00594-2)
Supplement: Supplementary file 1 — Supplemental Figure 1: Regulators Questionnaire (PDF 275 kb) [file 43441_2023_594_MOESM1_ESM.pdf]

# Mixed Reality Inspections Pilot



**Q1**

## Overall, how would you rate this experience in terms of its usefulness as an inspection tool?

Answered: 5 Skipped: 0

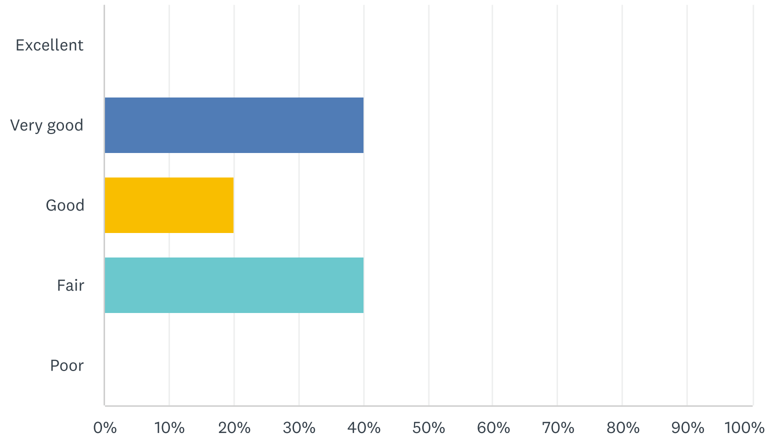


| ANSWER CHOICES | RESPONSES | |
|---|---|---|
| Excellent | 0.00% | 0 |
| Very good | 40.00% | 2 |
| Good | 20.00% | 1 |
| Fair | 40.00% | 2 |
| Poor | 0.00% | 0 |
| **TOTAL** | | **5** |

**Q2**

## What did you like about the use of the mixed reality tool in terms of its usefulness as a substitute for an on-site presence?

Answered: 5 Skipped: 0

The ability to inspect remotely and collaborate with the inspector/investigator on site. The potential to reduce traveling for inspectors/investigators and reduce regulatory burden for the manufacturing site. The potential to increase collaboration between regulatory authorities, harmonization and common understanding of GxP guidelines

9/10/2022 07:04 AM

Enables the viewer to see exactly what the person wearing the Halolens is seeing. Viewer can ask the individual to look certain directions in order to get a real time 360 degree view. Great microphone.

9/8/2022 09:59 AM

It can enable several countries and inspectors to participate in a single inspection thus harmonising the inspection process

**Q3**

## What did you dislike about the use of the mixed reality tool in terms of its usefulness as a substitute for an on-site presence?

Answered: 5    Skipped: 0

Technology limitations (connectivity, picture resolution, viewable area, sound)

9/10/2022 07:04 AM

Poor audio quality at the pre-inspection briefing. We were only hearing the person wearing the device

9/9/2022 09:22 AM

Requires strong WiFi to connect the Halolens. Connectivity interrupted while traveling throughout the facility until WiFi signal was reestablished. Ability to select tabs/screens within the Halolens appeared to be very precise and challenging for some users. Would require education and experience for the device wear prior to live streaming. Need to optimize battery life as the device died before the tour was completed. Potential safety concern if worn while walking throughout a facility. Is there an opportunity to optimize the field of view?

9/8/2022 09:59 AM

It is largely dependent on the skills, competence and ingenuity of the inspectors on the ground

**Q4**

## How could we improve the use of mixed reality during inspection?

Answered: 5    Skipped: 0

Establish principles for collaboration and interaction between on site inspectors/investigators and remote participants Improve the technology

9/10/2022 07:04 AM

Pre Review of quality document

9/8/2022 09:59 AM

The communication system needs to be improved to ensure that the entire conversation between the inspector and the inspected is conveyed. Additionally, 360 degrees cameras should be considered to illuminate the entire area during the inspection.

## Q5

# How likely are you to use the mixed reality device during inspection, if made available?

Answered: 5 Skipped: 0

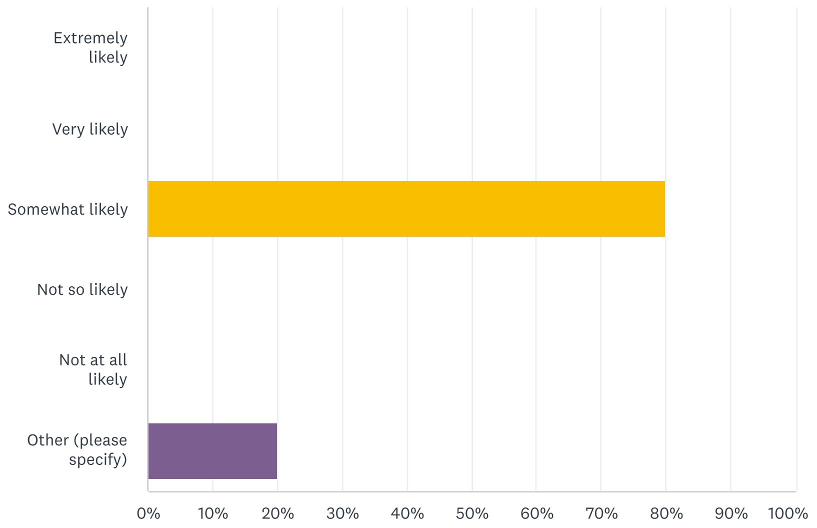


| ANSWER CHOICES | | RESPONSES | |
|---|---|---|---|
| Extremely likely | | 0.00% | 0 |
| Very likely | | 0.00% | 0 |
| Somewhat likely | | 80.00% | 4 |
| Not so likely | | 0.00% | 0 |
| Not at all likely | | 0.00% | 0 |
| Other (please specify) | Responses | 20.00% | 1 |
| **TOTAL** | | | **5** |

## Q6

# Other comments/questions?

Answered: 5 Skipped: 0

As suggested during the last virtual meeting, battery life must be prolong, Auditee readiness and preparedness must be ascertained, a liaison personnel between the onsite and offsite audits is also crucial.

9/9/2022 09:22 AM

Nothing additional

9/8/2022 09:59 AM

The connection was not always stable during the mock inspection, which was in USA, raising fears on what would happen in less resourced countries with lower internet bandwidth

**Q7**

## If you received all documents you requested before and during the inspection, could you write a GMP report based on virtual participation using the mixed reality device rather than participating on-site?

Answered: 5 Skipped: 0

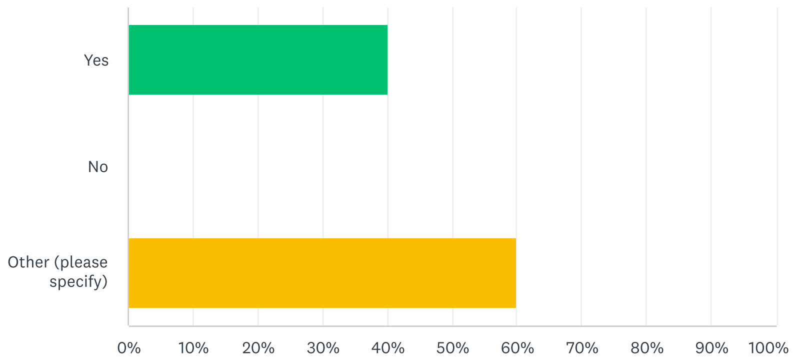


| ANSWER CHOICES | | RESPONSES | |
|---|---|---|---|
| Yes | | 40.00% | 2 |
| No | | 0.00% | 0 |
| Other (please specify) | Responses | 60.00% | 3 |
| TOTAL | | | 5 |

Powered by SurveyMonkey

Check out our sample surveys and create your own now!

Share Link https://www.surveymonkey.com/re COPY Share Tweet Share

5 responses
